# Epithelial-mesenchymal transition in prostate cancer: an overview

**DOI:** 10.18632/oncotarget.15686

**Published:** 2017-02-25

**Authors:** Micaela Montanari, Sabrina Rossetti, Carla Cavaliere, Carmine D'Aniello, Maria Gabriella Malzone, Daniela Vanacore, Rossella Di Franco, Elvira La Mantia, Gelsomina Iovane, Raffaele Piscitelli, Raffaele Muscariello, Massimiliano Berretta, Sisto Perdonà, Paolo Muto, Gerardo Botti, Attilio Antonio Montano Bianchi, Bianca Maria Veneziani, Gaetano Facchini

**Affiliations:** ^1^ Progetto ONCONET2.0, Linea Progettuale 14 per L'implementazione della Prevenzione e Diagnosi Precoce del Tumore alla Prostata e Testicolo, Regione Campania, Italy; ^2^ Department of Molecular Medicine and Medical Biotechnologies, University of Naples “Federico II”, Naples, Italy; ^3^ Department of Onco-Ematology Medical Oncology, S.G. Moscati Hospital of Taranto, Taranto, Italya; ^4^ Division of Medical Oncology, A.O.R.N. dei COLLI “Ospedali Monaldi-Cotugno-CTO”, Naples, Italy; ^5^ Pathology Unit, Istituto Nazionale Tumori “Fondazione G. Pascale”, IRCCS, Naples, Italy; ^6^ Radiation Oncology, Istituto Nazionale per lo Studio e la Cura dei Tumori ‘Fondazione Giovanni Pascale’, IRCCS, Naples, Italy; ^7^ Department of Uro-Gynaecological Oncology, Division of Medical Oncology, Istituto Nazionale Tumori ‘Fondazione G. Pascale’, IRCCS, Naples, Italy; ^8^ Department of Uro-Gynaecological Oncology, Division of Urology, Istituto Nazionale Tumori ‘Fondazione G. Pascale’, IRCCS, Naples, Italy; ^9^ Department of Medical Oncology, CRO Aviano, National Cancer Institute, Aviano, Italy; ^10^ Scientific Directorate, Istituto Nazionale Tumori ‘Fondazione G. Pascale’, IRCCS, Naples, Italy; ^11^ Directorate-General for Management, Istituto Nazionale Tumori ‘Fondazione G. Pascale’, IRCCS, Naples, Italy

**Keywords:** prostate cancer, epithelial-mesenchymal transition, androgen receptor, TGF-β signaling, EGF/EGFR

## Abstract

Prostate cancer is a main urological disease associated with significant morbidity and mortality. Radical prostatectomy and radiotherapy are potentially curative for localized prostate cancer, while androgen deprivation therapy is the initial systemic therapy for metastatic prostate disease. However, despite temporary response, most patients relapse and evolve into castration resistant cancer.

Epithelial-mesenchymal transition (EMT) is a complex gradual process that occurs during embryonic development and/or tumor progression. During this process, cells lose their epithelial characteristics and acquire mesenchymal features. Increasing evidences indicate that EMT promotes prostate cancer metastatic progression and it is closely correlated with increased stemness and drug resistance.

In this review, we discuss the main molecular events that directly or indirectly govern the EMT program in prostate cancer, in order to better define the role and the mechanisms underlying this process in prostate cancer progression and therapeutic resistance.

## INTRODUCTION

Prostate cancer (PCa) is the second most common cause of cancer-related deaths in male population [1]. Surgery represents a valid tool for the treatment of localized prostate cancer. Nevertheless, nearly all prostate cancer-associated mortality is caused by distant metastasis. The main therapeutic option for advanced prostate tumors treatment depends on androgen deprivation therapy (ADT) with limited clinical outcomes. Although the initial response rate is high, the therapeutic benefits are short-lived, and, within 18-24 months, the treated patients progress to metastatic castration-resistant prostate cancer (mCRPC), an androgen-insensitive disease, for which curative therapy is not available [2]. Therefore, the CRPC represents a critical demanding task for the prostate cancer cure. A greater comprehension of the molecular apparatus involved in PCa distant metastasis formation and metastatic castration-resistance is of critical importance to develop novel therapeutic approaches.

Recently, several evidences of epithelial-mesenchymal plasticity playing a role in both PCa metastatic progression and treatment resistance have been reported [3,4]. Epithelial-mesenchymal transition (EMT) has been initially defined as a complex molecular process required during the embryonic development for morphogenetic changes [5]. Therefore, epithelial cells heavily depend on a very fine tuned and highly regulated EMT program, which converts them into a mesenchymal state. Different factors, including microenvironment molecules able to accommodate cellular and tissue growth in normal or altered conditions, along with epithelial and stromal settings, are then required for achieving this program.

Sequential arrangements of adherent junctions, desmosomes and tight junctions allow epithelial cells to be strictly in contact with their immediate environment and also with their axis of polarity. In contrast, mesenchymal cells are loosely structured within a three-dimensional extracellular matrix, which also includes connective tissues [6]. The changes occuring in stroma, together with microenvironment transformation, contributes to create a compartment, where morphological effectors can affect the integrity and vitality of the tissues.

Recently, several articles have been demonstrated the EMT involvement in cancer progression and in metastasis formation, underlining that EMT is a significant event, responsible for triggering metastatic process [7,8]. In tumors, local cancer environment epithelial cells must transiently turn into a mesenchymal state for which these neoplastic epithelial cells take over the evolutionary conserved EMT process. Moreover, during EMT the cellular properties (cell-cell adhesion and cell polarity) of polarized epithelial cells are lost while an invasive, well-defined mesenchymal phenotype takes place as a result of multiple important features promoting metastatic development. Coordinated lost and gain of different epithelial (β-catenin, E-cadherin) and mesenchymal (vimentin, N-cadherin, fibronectin, and α-smooth muscle actin) markers, respectively, enhanced migratory and invasive abilities, increased apoptosis resistance, and extended production of extracellular matrix elements have been shown in human tumor samples and have been correlated to metastatic progression [9,10].

EMT typically occurs at the tumor-stroma interface, due to paracrine signaling molecules [11]. Different pivotal pathways, such as receptor tyrosine kinases, TGF-β/canonical and non-canonical SMAD signals, are activated within tumor cells. Importantly, by activating specific transcription factors (e.g. Snail, Twist and Slug), they cause the transcriptional repression of E-cadherin, a defining event in EMT [9, 11].

There are considerable data suggesting that EMT contributes to PCa progression and metastasis. In this review we summarize and discuss the main molecular events that directly or indirectly govern the EMT program in PCa, in order to better define the role and the mechanisms of this process in PCa progression and therapeutic resistance.

## EMT IN PROSTATE CANCER: ROLE OF ANDROGENS AND ANDROGEN-RELATED SIGNALING

Recently, the importance of EMT in tumor biology has evoked great interest in the scientific audience. Particularly, it has been reported that EMT play a pivotal role in promoting tumor metastasis, conferring cancer stem cell abilities, and mediating drug resistance in several preclinical model of different cancers [12–15], where the expression of EMT related molecules has been found to be well correlated with high grade tumors especially those that have poor prognosis [16].

Recently, many studies have demonstrated the presence of EMT-like states in PCa, suggesting its involvement in prostate cancer development and metastasis [17, 18]. The main players responsible for EMT in prostate need to be elucidated, and the effect of androgen deprivation therapy on EMT is still unclear.

Prostate is an androgen-dependent tissue that requires androgenic and androgen receptor (AR) signaling axis for normal functioning [19]. Androgens, whose action in adult males promotes the cell survival of secretory epithelial cells (those primarily transformed in tumor development), exert their functions through differential steps consisting of testosterone testicular synthesis, its transport to target tissues, and its reduction to 5α-dihydrotestosterone (DHT), mediated by 5α-reductase enzyme. Responsible for the physiological growth and development of prostate gland, and for the development and progression of prostate tumors, androgens display their biological activity by binding and consequently activating the androgen receptor (AR) [20]. However, deregulated androgen signaling is one of the most important factors in PCa progression and its pivotal role in modulating androgen-mediated EMT induction has been suggested, even if the mechanistic explanations regarding how the interplay between AR signaling and EMT-related transcription factors expression modulates EMT in PCa, needs to be further investigated.

Androgens are able to affect EMT of PCa epithelial cells. Here, they suppress the expression of E-cadherin, a calcium-dependent cell-cell adhesion molecule responsible for the formation of epithelial adherent junctions, and activate the expression of mesenchymal markers [21]. Interestingly, in PCa epithelial cells, the EMT pattern can be induced by androgens through the activation of Snail, leading to a relevant modification in PCa cell migration and invasion, by possibly eluding TGF-β functional involvement, and contributing to PCa metastatic behaviour [22]. Nevertheless, data regarding the androgens function in the control of EMT are conflicting. Although some reports have been demonstrated that androgens and AR play an active role in inducing EMT [22, 23], others have shown that EMT induction is due to androgen deprivation [24, 25] or low AR content [26] both in *in vivo* and *in vitro* prostate cancer settings. Moreover, androgen withdrawal promotes EMT in both normal and tumor prostate tissues, conferring a mesenchymal phenotype, despite differences in patient ages and tumor grades [27, 24]. Different *in vitro* and *in vivo* studies have suggested the effect of androgen deprivation on N-cadherin, E-cadherin, or vimentin expression, as well as a link between increased N-cadherin, androgen deprivation and metastasis formation in both human specimens and LNCaP xenografts [28, 29]. Interestingly, in the LNCaP cell line the expression of both N-cadherin and ZEB1, a EMT direct regulator, is enhanced by androgen deprivation and is associated with chemotherapy resistance [24, 29]. ZEB1, a transcription factor whose expression has been reported greatly increased after chemical or physical castration in LuCaP35 normal prostate xenograft model, human prostate specimens and LNCaP cell line, by potentially functioning as AR transcriptional suppressor, intervenes in EMT induced by androgen deprivation through a bidirectional negative feedback loop with androgen receptor, revealing a potentially important consequence of a standard-of-care treatment for PCa [24]. On the contrary, ZEB2 expression seems to be positively regulated by androgens in LNCaP cells; moreover, in PC3 and DU145 cells the inverse relationship between these two factors results in a decrease of invasion and migration properties of AR overexpressing PC3/DU145 cell, induced by E-cadherin (a ZEB2 transcriptional target) expression increase [30], leading to the hypothesis that these events occur as a consequence of ZEB2 reduced levels/activity in these androgen independent cells. Interestingly, miRNAs are involved in the post-transcriptional modulation of ZEB2 expression mediated by AR [31]. miR200 is responsible for the control of EMT by targeting ZEB proteins. Higher levels of miR200a/miR200b were found in LNCaP cells. In addition, in these cells the silencing of AR determines a reduction of miR200a/miR200b levels prompting to the hypothesis that androgen receptor may positively regulates the expression of miR200 [32]. However, a ZEB-miR200s double-negative feedback loop, responsible for regulating EMT phenotype stability and interchangeability, exists [33,34].

In LNCaP cells androgen withdrawal, along with the activation of PI3K-AKT and ERK pathways, leads to neuroendocrine differentiation (NED), which is correlated with PCa progression to a hormone refractory state, and poor prognosis [35–38]. In prostatic carcinoma, during neuroendocrine differentiation prostate cancer cells shift to neuroendocrine-like cells, characterized for expressing typical neuroendocrine markers (e.g. chromogranin A and neuron specific enolase) [36]. These neuroendocrine cells secrete factors that exert mitogenic responses on adjacent cells and promote androgen independent growth of PCa cells [38]. In LNCaP cells Snail regulates NED induced by either androgen deprivation or Snail over-expression. Therefore, by inducing both NED and EMT in these androgen-dependent cells, Snail can provide a tight connection between these two main events [39].

AR maintenance is then necessary for EMT regulation since its loss favors EMT in androgen-independent PCa cells. In addition, in the context of prostate tumor microenvironment, a role for AR signaling in prostate fibroblasts in promoting prostate epithelial cell proliferation [40], as well as in mediating the epithelium-stroma functional interaction, and then favoring the EMT events leading to metastatic disease, has been proposed [41]. Thus, the control of AR expression as well as its co-effectors and downstream players may have significant effect on prostate cancer metastasis [42].

## EMT IN PROSTATE CANCER: ROLE OF ESTROGENS AND ESTROGEN-RELATED SIGNALING

Interestingly, in the past years, a role for estrogen signaling in prostate cancer progression has been reported [43, 44]. Estrogens can directly and indirectly affects the development and homeostasis of prostate gland; along with their receptors, they can play a pivotal role in prostate tumor initiation and progression. Prostatic estrogen receptor alpha (ER-α) and -β (ER-β), whose expression patterns progressively differ during PCa progression, directly mediate the estrogens effect in this disease [45]. After binding with estrogen or estrogen-like compounds, ER-α and -β can homo- or heterodimerize and in the nucleus activate different signaling pathways [46]. On the other hands, the estrogen receptors can also interfere with androgen synthesis by repressing the hypothalamic-pituitary-gonadal (HPG) axis and, in turn, the direct effect on testis [47]. Then, due to the estrogen receptors presence in prostate, estrogens may directly function on prostate multiple sites. However, the role of ERs in the neoplastic events leading to PCa formation and progression still needs to be elucidated.

In normal prostate, ER-α and -β and their relative variants have very different expression patterns [48–50]. ER-α is primarily expressed in prostatic stroma where it displays indirect effects on prostate epithelia [51]. Instead, ER-β expression is found to be high in the gland epithelium, where ER-β is responsible for regulating its proliferation and differentiation [51]. In prostate cancer, methylation of ER-α gene leads to its silencing, loss of ER-α transcription and ER-α protein [52, 53]. Interestingly, in several PCa cell lines as well as in hormone refractory tumors and metastasis, ER-α expression has been observed suggesting it re-appears as cancer progresses [51, 54]. However, prostate cancer stromal ER-α was reported to interact with cancer-associated fibroblasts (CAFs) to inhibit PCa invasion through the selective up-regulation of thrombospondin 2 and down-regulation of matrix metalloproteinase 3 suggesting a protective role of stromal ER-α in prostate cancer progression [55].

In adult prostate gland ER-β represents the major estrogen receptor expressed; however, its role is not yet clearly established. ER-β seemed to act as a suppressive player of proliferation process, stimulating the differentiation of adult prostate epithelial cells [56,57]. In hormone refractory prostate cancers and in high Gleason grade prostate carcinomas, ER-β expression was found to be decreased, supporting the contention that loss of ER-β correlates with disease progression and that could be considered as a prognostic factor of prostate cancer [58, 59]. On the other hand, high ER-β protein levels have reported to be correlated to a worse prognosis in patients affected by this disease [60]. Interestingly, ER-β, when activated by 5α-androstane-3β-17β-diol, maintains the epithelial characteristics and represses the acquirement of mesenchymal traits and invasive abilities in prostate cancer cells [61]. Under these conditions, in aggressive and poorly differentiated high Gleason grade PCas, ER-β promotes the degradation of HIF-1α (hypoxia inducible factor 1 alpha subunit), a crucial EMT player, and downregulates VEGF (vascular endothelial growth factor), the canonical HIF target. Loss of ER-β therefore increases VEGF production, which employs neuropilin-1, a VEGF receptor, in driving EMT by promoting the nuclear localization of Snail1, through the inactivation of glycogen synthase kinase 3β (GSK3β), a critical regulator of Snail1 stability and localization [61]. In addition, due to deletion of PTEN that represents one of the most frequent prostate cancer genetic alteration, prostate tumorigenesis is promoted by BMI-1-mediated repression of ER-β that enables the HIF-1α/VEGF signaling altered in PCa cells [62]. Interestingly, ER-β2 and ER-β5 variants, which have tumor-promoting actions, are able to stabilize HIF-1α and allow the expression of hypoxic genes in prostate cancer [63].

The capability of HIF to promote EMT might account for the common association of increased HIF levels and intratumoral hypoxia with a poor prognosis in different cancers, including prostate cancer. By suppressing ER-β and stimulating HIF-1α-mediated VEGF expression, hypoxia can promote the acquisition of mesenchymal features in PCa cells. Since ER-β is sensitive to changes in tumor microenvironment, hypoxic conditions and TGF-β1 signaling can reduce ER-β levels and alter its action, favoring PCa progression.

Further studies, aimed to disclose all molecular mechanisms of estrogen signaling during this disease, are then needed to provide new insights into prostate cancer biology and novel hormonal therapeutic approaches for prostate cancer treatment.

## ROLE OF TGF-β SIGNALING IN EMT INDUCTION OF PROSTATE CANCER CELLS

Among the molecular factors involved in EMT induction, the TGF-β (transforming growth factor β) represents one of the main player in inducing this process. In normal and premalignant cells, TGF-β functions as an effective tumor suppressor, promoting apoptosis and cell differentiation [64]. Nevertheless, during tumor progression, its suppressive properties are lost, and, consequently, cells display a proliferative phenotype, initiate immune evasion, growth factors production and EMT [65]. In tumors, cancer cells bypass TGF-β suppressive functions, either through directly inactivation of TGF-β receptors or through repressing specific downstream elements, which maintain the epithelial phenotype [66].

Smad-dependent and Smad-independent pathways are responsible for TGF-β induced EMT [66]. Upon activation by the binding of an active TGF-β ligand, TGF-β dimers cause the formation of heteromeric complex between specific TGF-β type II and type I receptors, which, in their phosphorylated form, propagate the signal into the cell by phosphorylating Smad2 and Smad3 proteins (its receptor substrates). In the nucleus, the phosphorylated Smads bind to Smad4 and form a protein complex which, facilitating its association with DNA-binding cofactors, then activate sequential transcriptional programs in response to TGF-β [66]. Smad-dependent pathway promotes TGF-β tumor suppressive roles, whereas the activation of Smad-independent pathways, along with the loss of TGF-β tumor suppressor roles, is responsible for its pro-oncogenic activity [67,68]. In prostate cancer, increased TGF-β expression is correlated with progression to advanced disease, with a prognostic value for survival [69], while loss of TβRI and TβRII is associated with poor prognosis [70].

Also, TGF-β family proteins communicate through non-canonical Smad-independent signaling, resulting in functionally different transcriptional outcomes. Ras-Raf-Erk mitogen-activated protein kinase (Erk/MAPK), Phosphotidylinositol-3kinase-Akt (PI3K/AKT) and JNK/p38 MAP kinase (JNK/p38MAPK) signaling pathways are TGF-β downstream effectors, each with distinct contributions to EMT [71]. TGF-β stimulation of Erk through Ras, Raf, and downstream MAPK is necessary in EMT, as Erk signaling controls genes involved in ECM-cell interactions and cellular motility [72]. Furthermore, the PI3K/AKT pathway represents an important player of TGF-β-mediated EMT; PI3K blockade leads to EMT inhibition, whereas forced expression can effectively disturb cellular junctions [72].

The phenotypic modification necessary for EMT likely occurs through non-Smad mediated TGF-β signaling, while TGF-β growth inhibitory functions are mediated by canonical Smad-dependent signaling [51]; however, a potential signaling synergy between non-Smad and Smad can occur, in turn inducing cellular plasticity and impacting EMT [73].

A role for TGF-β-induced EMT has been suggested in the development of benign prostatic hyperplasia (BPH) where stromal cells are able to induce EMT, possibly through secreting TGF-β1 and activating its signaling [74, 75]. Stromal cells greatly influence the tumorigenesis in adjacent epithelia. Intraepithelial neoplasia in prostate cancer, combined with an increase in the amount of stromal cells, represents the direct consequence of the loss of TGF-β responsiveness in fibroblasts. Thus, TGF-β pathways in fibroblasts promotes prostate cancer by modulating the adjacent epithelial cell growth and its oncogenic ability [76, 77]. In addition, in prostate cancer, TGF-β determines the nuclear accumulation of nuclear factor-kappa B (NF-κB), and for the morphological changes occurring towards a mesenchymal phenotype [78, 79]. However, EMT like features have been shown to be blocked by the inhibitor of NF-κB demonstrating that NF-κB is an important intermediary of TGF-β mediated EMT in PCa [79]. Current information related to TGF-β induced EMT in PCa are well documented albeit a conclusive evidence of EMT from human tissues and the role of TGF-β (stromal and/or epithelial) in EMT induction in PCa is still missing.

TGF-β is able to regulate AR gene targets in a close crosstalk with androgens, with AR-dependent outcomes [80, 41]. The crosstalk between TGF-β signaling and androgen axis may represent a crucial element for EMT expression. Changes in cytoskeleton reorganization induced by androgens may provide active movements that assist prostate cancer cells migration and metastasis. In castrate-resistant prostate tumors a dynamic AR/β-catenin crosstalk leads to their increased nuclear co-localization and interaction [81]. The binding of β-catenin to cadherin represents a requirement for adhesion; however, its dissociation from the complex formed with cadherin enables it to reach the nucleus, where β-catenin functions as a transcription factor, and controls genes responsible for EMT in prostate cancer. Interestingly, in prostate cancer epithelium EMT can be induced through a different mechanism involving β-catenin activation by androgen signaling [22].

Conserved zinc finger transcription factors, controlled by upstream TGF-β activities, have been reported to induce EMT functions [82,83]. Activated Snail, an EMT key transcription factor whose expression increases during PCa progression [84], represses E-cadherin expression, which is one of the hallmarks of EMT, in several cancer cell types [85–88]. Snail ectopic expression alone can cause EMT and improve cell motility in cancer cells [87, 89]. Moreover, its knockdown partially reverses EMT and enhances cell motility [89, 90].

In TGF-β-responsive LNCaP-TβRII cells, Snail expression significantly increases after treatment with 5α-dihydrotestosterone alone or in combination with TGF-β indicating that androgens, by avoiding the effect evoked by TGF-β, can independently promote EMT [22]. In prostate cancer microenvironment Snail is able to interact not only with TGF-β but also with epidermal growth factor (EGF) in downregulating the expression of HLA-I (human leukocyte antigen class I), which influences prostate cancer progression [91]. Moreover, in prostate cancer cells Snail knockdown significantly reverses HLA-I downregulation induced by TGF-β and EGF, confirming that Snail is crucial for this process [91].

Genetically engineered mouse models provide direct evidence to support TGF-β role in driving prostate tumor metastasis. TGF-β signaling *in vivo* disruption was shown to accelerate the pathologic malignant prostatic phenotype of the TRAMP mouse model by altering prostate growth and inducing EMT [92].

The complex signaling pathways involving Smad and non-Smad proteins can play a pivotal role in orchestrating EMT and metastatic responses. The identification of new important players contributing to prostate tumor progression as well as additional TGF-β signaling effectors, useful either prognostically or diagnostically for characterizing EMT, may allow the development of new therapeutical tools for this urological disease.

## EGF SIGNALING AND CROSSTALK IN PROSTATE CANCER

EGF represents a crucial mitogenic factor able to control physiological prostatic functions [93]. However, following the binding to EGF receptor (EGFR), it stimulates cellular growth, vitality, migration and metastatic abilities of different tumors, including prostate cancer [94]. EGF and EGFR are aberrantly expressed in both androgen independent and metastatic PCa, which possess heightened EMT related features, and are closely associated with aggressive phenotype, poor clinical prognosis, high Gleason score, reduced survival rate [95–97], then contributing to castrate resistant PCa and progression to metastasis. Interestingly, recent evidences have demonstrated an important role of EGF/EGFR signaling in inducing epithelial-mesenchymal transition and thereby promoting cell motility in epithelial cells from several tumor types [98–101]. In carcinomas EMT marker expression and EGFR over-expression are almost simultaneous and this event is associated with enhanced metastasis [102]. In prostate cancer, the acquired mesenchymal spindle-like cell structure, the increased expression of fibronectin and N-cadherin, and the E-cadherin concurrent decrease, represent the specific traits of EMT induced by EGF [103]. In PC-3 cells EGF is able to induce EMT by promoting EMT itself and PC-3 cells motility through a protein kinase C (PKC)/GSK-3β/Snail signaling pathway. EGF promotes the stability of upregulated Snail by inducing PKC activation and consequently preventing GSK-3β phosphorylation activity, which in turn decreases Snail ubiquitination and increases its transcription [103]. The PI3K/AKT pathways has been reported to modulate the activity of GSK-3β. Following its activation by PI3K phosphorylation, AKT phosphorylates GSK-3β and inhibits its activity [104]. EGF induced EMT was found to be mainly dependent on Akt activation, as inhibition of Akt signaling abolished EGF driven EMT in PCa cell lines [105, 106]. However, although EGFR overexpression and EGF signaling constitutive activation in prostate cancer are associated with poor prognosis, the exact role played by EGF/EGFR in the progression and development of this disease still needs to be elucidated. Recent data underscore the importance of the heat shock protein 27 (Hsp27) in EGF-mediated EMT in prostate cancer suggesting that EGF-induced EMT requires Hsp27 and involves β-catenin/Slug signaling pathway [107]. Hsp27 is a molecular chaperone, whose expression is upregulated following different cellular stresses, which plays a pivotal role in controlling cellular migration and invasion [108]. Highly expressed in prostate cancer [109], Hsp27 is correlated with aggressive cancer behaviour and metastatic potential [110], and represents a prognostic marker of poor clinical outcome [111]. Its increased expression correlates with prostate cancer progression to castration-resistant disease [112] and it is essential for EGF-promoted cell migration and invasion, as well as for EMT markers expression [108], AKT and GSK3β phosphorylation induced by EGF, and consequently for β-catenin stabilization [107]. These latter events, in turn, allow β-catenin nuclear translocation, EMT transcription factor Slug consequent activation and, finally, prostate cancer migration and invasion [107]. Hsp27 may act as an inducer of EMT activated by EGF; in addition, in prostate cancer it plays a crucial role in controlling both E-cadherin expression modulation mediated by β-catenin and EMT transcriptional regulation. Interestingly, in high grade PCa Hsp27 expression correlates with an increase in the expression of EMT player Twist [113]. A strong correlation between both Hsp27 and EGF and advanced prostate cancer, as well as with EMT through IL-6/STAT3/Twist pathway, has been reported [113,114]. Furthermore, EGF favors PCa progression through a ROS/STAT3/HIF-1α/Twist1/N-cadherin signaling pathway [114]. Twist1 is a highly conserved, basic helix-loop-helix (bHLH) domain-containing transcription factor able to induce metastasis [115], and also EMT. In prostate cancer a key role for Twist1 during EGF-induced EMT and tumor invasion has been proposed since it acts as a crucial downstream mediator of these events. In prostate cancer EGF stimulates tumor progression via the coordinated ROS production, the phosphorylation of STAT3 (required for EGF-induced upregulation of HIF-1α) and the consequent induction of HIF-1α/Twist1/N-cadherin signaling pathway [114].

Interestingly, EGF can interact with Src tyrosine kinase pathway in the induction of EMT in TMPRSS2-ERG positive prostate cancers [116]. TMPRSS2-ERG is a fusion transcript that emerges as prostate cancer cells specific biomarker; when overexpressed, it plays a pivotal role in prostate cancer transformation, EMT and invasion [117–120]. ERG (ETS-related-gene), which represents the most commonly over-expressed and translocated oncogene in PCa cells [121], arises from genomic fusion between androgen-regulated TMPRSS2 gene promoter upstream sequences and ERG coding sequences [122, 120]. Despite androgen represents the major driver of TMPRSS2-ERG overexpression in primary tumors, EGF and Src can modulate the expression of TMPRSS2-ERG in PCa cells through a different pathway where they upregulate ERG expression via miR-30 modulation [116]. This novel crosstalk between the androgen/AR and EGF/Src signaling pathways, by connecting EGF and Src signaling pathways to ERG induction and EMT, via miR-30b silencing, assumes a relevant role in castration-resistant PCa, where the androgen receptor is abnormally activated despite androgen absence.

## EMT AND CANCER STEM /PROGENITORS CELLS IN PROSTATE CANCER

Recently, EMT has been linked to stem cell phenotype [123] since EMT acquires stem cell-like features, such as self-renewal and slow proliferation [124,125]. After androgen-deprivation therapy, both cancer cells with EMT markers and those with cancer stem cells (CSCs) markers increase in human prostate cancer specimens [126,127]. Stem-like stem/progenitors cells acquire more complete EMT molecular characteristics and exhibit more aggressive abilities. Specifically, prostate CSCs display increased EMT markers as well as increased tumorigenesis, migration and invasion ability [128]. The molecular mechanism underlying EMT and regulation of stemness is still not clear. Cell signaling pathways including PI3K/AKT, EGF/EGFR, STAT3/5, Wnt/β-catenin, have been shown to sustain stem cell growth and self-renewal further confirming the important function played by stem cell/progenitors cells in PCa progression [129, 130]. EMT activator Twist1 can promote both stemness and EMT properties [131, 132], providing a connection between CSCs and EMT. Moreover, in PCa the PI3K/AKT/mTOR signaling activation induces EMT and enhances CSCs phenotype, responsible for the associated radioresistance of this disease [133]. Interestingly, AR, whose expression is low in prostate cancer stem cell/progenitors cells, plays a negative role in EMT of these cells, by controlling AKT pathway [134].

PCa cancer stem/progenitor cells and metastasis are inhibited by miR-34a through the direct repression of CD44, a major adhesion molecule of the extracellular matrix, which is also a marker of CSCs [135]. CD44 plays an important role in inducing EMT and/or in maintaining the mesenchymal phenotype in PCa [136,137]. As a marker of cancer stem/progenitor cells, CD44, by promoting EMT and metastasis in PCa carcinogenesis, may serve a new potential prostate cancer therapeutic approach. Furthermore, CD44 high expression level was associated with biochemical recurrence and distant metastasis, making CD44 a marker of poor prognosis in prostate cancer [136]. In addition, CD44+ stem-like cell population increases in prostate cancer cells following ADT–induced TGF-β signaling activation leading to de-differentiated phenotype of prostate cancer cells [137]. CD44^+^ cancer stem/progenitor cells, in response to TGF-β signaling, not only can initiate the EMT but also can regulate the mesenchymal phenotype in prostate cancer cells.

However, additional signaling pathways are responsible for regulation of stemness and EMT of PCa cells. Overexpressed N-cadherin, a marker of ongoing EMT, achieves EMT, stemness promoting function and metastatic ability by activating the ErbB signaling in PCa cells, further supporting the hypothesis that N-cadherin could be a promising target in PCa therapy [138]. Abnormal expression of N-cadherin has been shown as crucial not only to metastasis, but also to castration resistance [28, 29], and associated with poor prognosis [138,139]. Simultaneous upregulation of N-cadherin and downregulation of E-cadherin have been found in more aggressive cultured PCa cell lines and in primary and metastatic prostate tumors [140–142]. Mice xenograft models treated with N-cadherin monoclonal antibody have displayed an inhibition of tumor growth and metastasis [28]. Recent study showed that miR-145 played a pivotal role in inhibiting migration, invasion, stemness, and EMT properties of prostate cancer cells via different targets [143, 144]. In gastric cancer N-cadherin is miR-145 direct target and promotes the invasion and metastasis cascade [145]. However, since N-cadherin promotes prostate cancer cells stemness features and EMT, a role for N-cadherin in mediating miR-145 function in the regulation of these events has been proposed [146].

It is well established that Wnt pathway has a developmental role in tissues and organisms. However, the aberrant activation of the Wnt/β-catenin pathway correlates with EMT features and also positively affects proliferation and invasiveness in PCa [147]. Interestingly, miR-320, a CD44+ prostate CSCs negative regulator, can inhibit the expression of β-catenin and repress CSCs via Wnt/β-catenin pathway inhibition [148]. In addition, some CSCs features (e.g. tumor-spheres formation, tumorigenicity, chemioresistance) are enhanced by downregulation of miR-320 [148].

In NOD/SCID IL2RY null mice Erismodegib (an inhibitor of Sonic hedgehog pathway) can inhibit the growth of human prostate CSCs and EMT by upregulating miR-200 and consequently inhibiting Snail, Slug and Zeb1 transcription factors, and preventing Bmi-1, which contributes to the self-renewal capacity of prostate CSCs, through miR-128 induction, respectively [149].

Then, many miRNAs, by regulating EMT, cancer cell migration and invasion, and CSCs properties may represent the common step combining EMT, CSCs, and drug resistance.

## CONCLUSIONS

The reappearance of the tumor acquired drug resistance along with some features of EMT and stemness have been responsible for the failure of traditional chemotherapy and/or androgen ablation treatment for the therapy of advanced/metastatic PCa. Thus, to prevent the metastatic spread, novel strategies are being used, which inhibit cancer cell proliferation, EMT and stem cell-like properties.

Altered miRNAs are correlated to prostate cancer initiation, progression and metastasis [150], and their aberrant expression can allow the progression to resistant prostate tumors [151]. In androgen independent prostate cancers miR−221/−222 and miR-125b are frequently upregulated promoting prostate cancers cells resistance to androgen withdrawal and androgen-independent cell growth [152, 153]. Furthermore, circulating miRNAs are correlated with PCa development and its progression to androgen-independent disease. Elevated expression of miR-141, miR-375 and miR-378 have been found in the serum of prostate cancers patients suggesting their potential use as PCa diagnostic biomarkers [154]. On the other hands, different miRNAs, capable of inhibiting cancer progression, are down-expressed in androgen independent prostate tumors, resulting in high and low levels of mesenchymal and epithelial markers, respectively, that can induce EMT, increase prostate cancer aggressiveness and resistance to androgen deprivation therapy [155].

Due to their advantageous *in vivo* stabilities and pharmacokinetics characteristics, small molecule drugs have emerged as favorable tool in the regulation of miRNAs and in potentially suppressing drug resistance in PCa [156, 157]. Since the modification of abnormal miRNAs levels can make resistant PCa cells more sensitive to small molecule drugs, the combined use of these innovative agents (such as small molecules and miRNAs) may represent a favorable approach for the therapeutic treatment of resistant prostate cancers.

**Figure 1:**
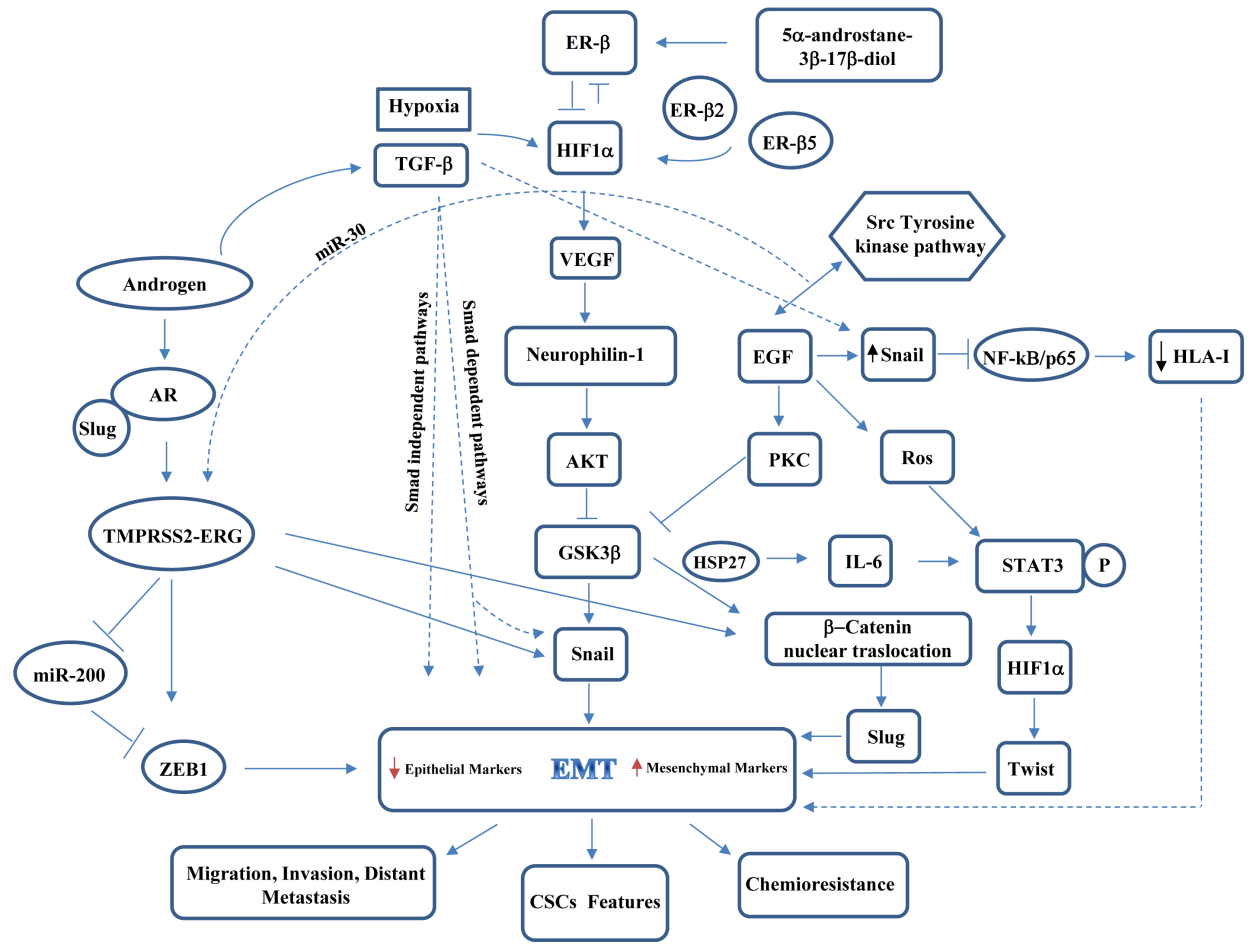
Schematic representation of main players and crosstalks governing EMT in prostate cancer

Current studies regarding prostate tumor microenvironment have highlight that signals arising from microenvironment can play a key role in governing EMT and highly influence cancer progression or clinical outcomes in PCa patients. Several key regulators and inducers, supported by their crosstalking signaling pathways and ligands, are then responsible for the transition to a dedifferentiated invasive phenotype (Figure 1). The detailed characterization of the crosstalks between EMT and castration resistance may offer the potential to identify novel prognostic or diagnostic markers as well as to develop new tailored therapeutic approaches for PCa management.
